# The Role of Disease Activity Score 28 in the Evaluation of Articular Involvement in Systemic Lupus Erythematosus

**DOI:** 10.1155/2014/236842

**Published:** 2014-11-03

**Authors:** Fulvia Ceccarelli, Carlo Perricone, Laura Massaro, Viviana Antonella Pacucci, Enrica Cipriano, Simona Truglia, Francesca Miranda, Francesca Romana Spinelli, Cristiano Alessandri, Guido Valesini, Fabrizio Conti

**Affiliations:** Lupus Clinic, Reumatologia, Dipartimento di Medicina Interna e Specialità Mediche, Sapienza Università di Roma, Viale del Policlinico 155, 00161 Roma, Italy

## Abstract

*Objectives*. To evaluate the application of Disease Activity Score 28 (DAS28) to assess joint involvement in Systemic Lupus Erythematosus (SLE).* Methods*. Sixty-nine SLE patients, complaining of joint symptoms, and 44 rheumatoid arthritis (RA) patients were enrolled. In SLE patients disease activity was assessed with SLEDAI-2K. DAS28 was calculated in all the patients.* Results*. Thirty SLE patients (43.5%) showed clinical signs of arthritis. Mean DAS28 was 4.0 ± 1.4, 22 patients (31.9%) had low disease activity, 29 (42.0%) moderate, and 18 (26.1%) high. We dichotomized SLE patients according to the presence (Group 1) or absence (Group 2) of articular involvement according to SLEDAI-2K: 56.3% of the patients of the second group had a moderate/high activity according to DAS28. We compared SLE patients with 44 RA patients (M/F 9/35, mean age 55.6 ± 14.5 years; mean disease duration 140.4 ± 105.6 months). No significant differences were found regarding the values of DAS28 between SLE and RA patients. On the contrary, the values of tender and swollen joint count were significantly higher in RA compared to SLE patients (*P* = 0.0002 and *P* = 0.0001, resp.).* Conclusions*. We suggest the use of the DAS28 in the assessment of joint involvement in SLE patients.

## 1. Introduction

Joint involvement is a frequent manifestation in patients affected by Systemic Lupus Erythematosus (SLE) and it occurs in up to 90% of patients at the onset or during the course of the disease [1].

The range of this involvement could be very wide: SLE patients can experience arthralgia, synovitis, with the presence of signs and symptoms of inflammation, or deforming arthropathy, with different degrees of severity and rarely erosive damage [1].

The traditional definition of “nonerosive SLE arthritis” has undergone substantial changes, due to the introduction of new imaging techniques with high sensitivity in the evaluation of bone surface [2–4].

In the light of the above-mentioned considerations, the assessment of joint involvement is a central topic in SLE management. So far, no specific and validated indices able to assess articular involvement in these patients are available. However, the global indices currently used to evaluate the disease activity in SLE patients include specific items in order to evaluate the presence of active articular manifestations. In the Systemic Lupus Erythematosus Activity Index 2000 (SLEDAI-2K), one of the most frequently used, arthritis is defined as “≥2 joints with pain and signs of inflammation (i.e., tenderness, swelling or effusion)” [5]. It is therefore clear that this index is not able to capture all the possible features of joint involvement in SLE patients and to evaluate their changing over time.

In the past years, the Disease Activity Score 28 (DAS28) has been validated and widely used in clinical trials and in routine clinical practice in patients with rheumatoid arthritis (RA). This is an easy to perform, standardized activity index that highly correlates with physician's and patient's global assessment and can classify disease activity into low, moderate, or high, as well as identify disease remission [6, 7].

Even if validated only for patients suffering from RA, DAS28 has been used in the monitoring of patients affected by other rheumatic conditions, characterized from polyarticular involvement, such as Psoriatic Arthritis (PsA) [8].

As the peripheral joint involvement of SLE patients shares some clinical characteristics with RA, the aim of the present study was to evaluate the application of DAS28 in the assessment of joint involvement in SLE, to correlate it with the global index SLEDAI-2K and to compare the results with those obtained in a group of RA patients.

## 2. Patients and Methods

During a 1-month period, all consecutive adult patients affected by SLE, diagnosed according to the 1997 ACR revised criteria [9], referring to the Lupus Clinic, Rheumatology Unit, Sapienza University of Rome, complaining of joint manifestations at the time of evaluation (at least one tender joint) were enrolled. In the same 1-month period, we enrolled as controls 44 consecutive RA patients, diagnosed according to the ACR/EULAR 2010 revised criteria [10], followed at the Rheumatology Unit, Sapienza University of Rome.

Clinical and laboratory data were collected in a standardized, computerized, and electronically filled form, which included demographics, past medical history with date of diagnosis, comorbidities, and previous and concomitant treatments.

The study was conducted according to the protocol and good clinical practice principles and the Declaration of Helsinki statements. All patients gave their informed consent and the study was approved by the local ethics committee.

### 2.1. Clinical and Laboratory Evaluation of SLE Patients

According to 1997 ACR revised criteria [9], we registered the presence of the following SLE manifestations.

*(i) Skin Involvement*. Malar Rash (fixed erythema, flat or raised, over the malar eminences, tending to spare the nasolabial folds), Discoid Rash (erythematous raised patches with adherent keratotic scaling and follicular plugging; atrophic scarring may occur in older lesions), and Photosensitivity (skin rash as a result of unusual reaction to sunlight, by patient history or physician observation).

*(ii) Oral Ulcers*. Oral or nasopharyngeal ulceration observed by physician.

*(iii) Serositis*. Pleuritis (convincing history of pleuritic pain or rubbing heard by a physician or evidence of pleural effusion) or Pericarditis (documented by electrocardiogram or rub or evidence of pericardial effusion).

*(iv) Kidney Involvement*. Persistent proteinuria >0.5 grams per day or > than 3+ if quantitation not performed or cellular casts (red cell, hemoglobin, granular, tubular, or mixed).

*(v) Neurologic Disorder*. Seizures (in the absence of offending drugs or known metabolic derangements, e.g., uremia, ketoacidosis, or electrolyte imbalance) or Psychosis (in the absence of offending drugs or known metabolic derangements, e.g., uremia, ketoacidosis, or electrolyte imbalance).

*(vi) Hematologic Disorder*. Hemolytic anemia with reticulocytosis or Leukopenia <4.000/mm^3^ on ≥2 occasions or Lymphopenia <1.500/mm^3^ on ≥2 occasions or Thrombocytopenia <100.000/mm^3^ in the absence of offending drugs.

*(vii) Immunologic Disorders*. Anti-DNA or Anti-Sm or positive finding of antiphospholipid antibodies on (1) an abnormal serum level of IgG or IgM anticardiolipin antibodies, (2) a positive test result for lupus anticoagulant using a standard method, or (3) a false-positive test result for at least 6 months confirmed by* Treponema pallidum* immobilization or fluorescent treponemal antibody absorption test.

SLE patients underwent peripheral blood sample collection and sera were stored at −20°C until assayed. Antinuclear antibodies (ANA) were performed by means of indirect immunofluorescence (IIF) on HEp-2 and anti-dsDNA by IIF on* Crithidia luciliae* in accordance with the manufacturer's instructions (Orgentec Diagnostika, Mainz, Germany). ENA (anti-Ro/SSA, anti-La/SSB, anti-Sm, anti-RNP), anticardiolipin (anti-CL, IgG, and IgM isotype), and anti-*β*2-glycoprotein I (anti-*β*2GPI, IgG, and IgM isotype) antibodies were performed by ELISA (Diamedix, Miami, FL, USA); lupus anticoagulant (LA) was assessed according to the guidelines of the International Society on Thrombosis and Hemostasis (Scientific Subcommittee on lupus anticoagulant/phospholipid-dependent antibodies) [11]. Complement C3 and C4 serum levels (mg/dL) were studied by means of radial immunodiffusion. The erythrocyte sedimentation rate (ESR) was determined with standard methods (mm/h, Westergren) both in patients with SLE and RA. Global disease activity was assessed in SLE patients with the SLEDAI-2K [5].

### 2.2. Joint Involvement Assessment

The same rheumatologist evaluated SLE and RA patients. Clinical evaluation included swollen and tender joint count (0–28) and global health (GH) on a visual analogue scale (0–100 mm). Disease activity score (28-joint count, four variables, ESR-based; DAS28) was calculated. According to DAS28 values, the clinical remission and the disease activity status were assessed [6, 7]. Specifically, the level of disease activity is defined as low (DAS28 ≤ 3.2), moderate (3.2 < DAS28 ≤ 5.1), and high (DAS28 < 5.1); finally, remission was defined as DAS28 values lower than 2.6 [6, 7].

### 2.3. Statistical Analysis

Statistical analyses were performed using Statistical Package for Social Sciences (SPSS 13.0, Chicago, IL, USA) and the MedCalc version 16.0 (MedCalc Software, Mariakerke, Belgium). Continuous data were presented as means with standard deviations (SDs) or medians with 95% confidence interval (95% CI), depending on the distribution of the data (tested with the Kolmogorov-Smirnov test). Histogram was used to visualise the distribution of the DAS28 score. Categorical data were presented as proportions.

Mann Whitney test was performed. Univariate comparisons between nominal variables were calculated using chi-square (*χ* ^2^) test or Fisher-test where appropriate. Pearson's and Spearman's tests were used to perform the correlation analysis where appropriate. Two-tailed *P* values were reported; *P* values less than or equal to 0.05 were considered significant.

## 3. Results

Sixty-nine SLE patients complaining of joint symptoms were enrolled (M/F 2/67, median age 43 years (range 23–72), median disease duration 120 months (range 1–420)). In Table 1 the main clinical and laboratory features of SLE patients are reported, both concerning the whole disease history and at the time of enrolment.

The mean ± SD SLEDAI-2K value registered in our cohort was 2.6 ± 2.5. At the time of the enrolment, all SLE patients had arthralgias, and 30/69 (43.5%) showed clinical signs of arthritis. Two patients had Jaccoud's arthropathy.

**Figure 1:**
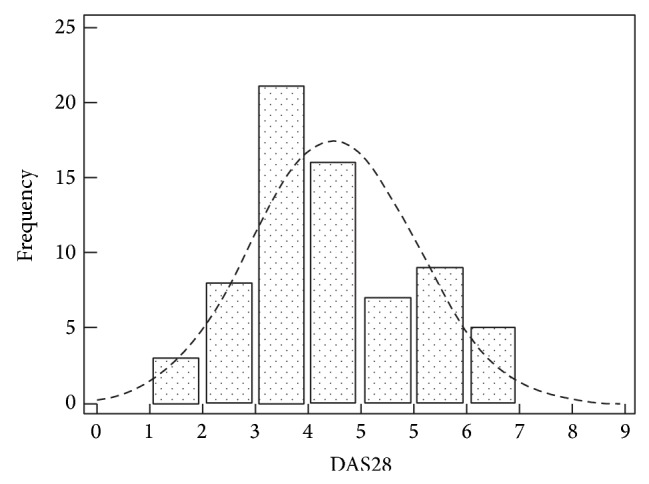
Estimates of central tendency and distributions of DAS28 in SLE patients.

The mean DAS28 value in SLE patients was 4.0 ± 1.4.  Figure 1 shows estimates of central tendency and distributions for DAS28; the index values were normally distributed (Kolmogorov-Smirnov test). The majority of patients showed DAS28 values between 3 and 5.

According to DAS28 values, 22 patients (31.9%) had low disease activity, 29 (42.0%) moderate disease activity, and 18 (26.1%) high disease activity; moreover 8 SLE patients (11.6%) were in remission (DAS28 < 2.6).

**Figure 2:**
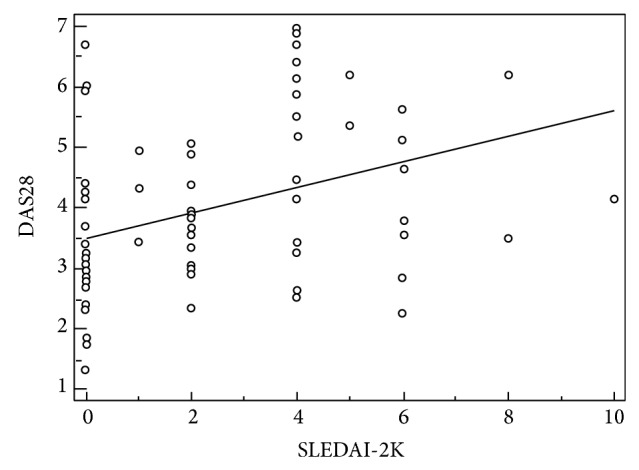
Correlation between DAS28 values and SLEDAI-2K (*r* = 0.4; *P* = 0.0006).

We identified a positive correlation between DAS28 and SLEDAI-2K values in patients with SLE (*r* = 0.4, *P* = 0.0006; Figure 2).

Moreover, we dichotomized SLE patients according to the presence (Group 1) or absence (Group 2) of articular involvement as defined by SLEDAI-2K (Table 2).

As expected, Group 1 patients showed significantly higher values in terms of SLEDAI-2K (*P* = 0.0001). No significant differences were registered between the two groups when considering DAS28 mean values, even in the presence of significant higher mean values of tender and swollen joint count, GH and ESR (*P* = 0.0001, *P* = 0.0001, *P* = 0.01, *P* = 0.01, resp.). Group 1 presented a statistically significant higher percentage of patients with DAS28 high disease activity, even though, in Group 2, only 16.7% of the patients showed a remission condition according to DAS28; conversely, 56.3% of patients in this group had a moderate/high activity.

**Figure 3:**
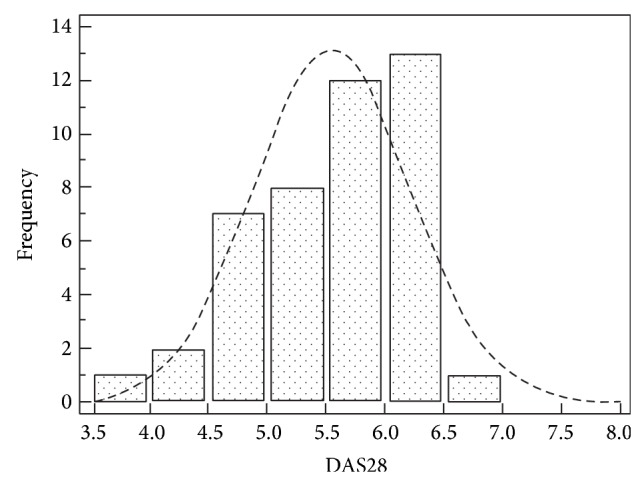
Estimates of central tendency and distributions of DAS28 in SLE patients.

Finally, we performed a comparison with a group of patients affected by RA patients (M/F 9/35, mean age 55.6 ± 14.5 years; mean disease duration 140.4 ± 105.6 months). RA patients showed a mean age significantly higher than SLE subjects (*P* = 0.0001). The mean DAS28 value registered in RA patients was 5.5 ± 1.2: Figure 3 shows estimates of central tendency and distributions for DAS28; the index values were normally distributed (Kolmogorov-Smirnov test).

No significant differences were found regarding the values of DAS28, ESR, and GH between SLE and RA patients. On the contrary, the values of tender and swollen joint count were significantly higher in RA patients compared with SLE (*P* = 0.0002 and *P* = 0.0001, resp.) (Table 3).

## 4. Discussion

In the present study, for the first time, we analyzed the application of the disease activity index DAS28 in a group of patients affected by SLE with joint involvement. Our analysis demonstrated that up to 50% of SLE patients without joint involvement as defined by SLEDAI-2K item showed a moderate/high disease activity according to DAS28 values. These results suggested a greater ability in the assessment of SLE-related articular manifestations, compared with the global activity index SLEDAI-2K.

SLE is a multifactorial autoimmune disease characterized by different pathogenetic mechanisms and by the development of a wide range of serum autoantibodies [12–15].

Besides the known role of the B cells, more recently the involvement of T Lymphocytes has been suggested [13, 14]. The clinical heterogeneity is the result of this complex pathogenetic mechanism: neuropsychiatric and renal involvement have been considered the most important manifestations in terms of severity and prognosis [16, 17]. The disease showed different degrees of severity and unpredictable flares and remission periods [18].

Despite the high prevalence in SLE patients, arthritis remains understudied and poorly understood. SLE articular involvement is characterized by a wide heterogeneity of manifestations, ranging from arthralgias, without any sign or symptom of inflammation, to deforming nonerosive reversible arthropathy (Jaccoud's arthropathy), finally to erosive arthritis, especially in patients with Rhupus [1–4].

Several global disease activity indices have been validated to assess patients affected by SLE. Among these, SLEDAI-2K and the British Isles Lupus Assessment Group (BILAG) index are the most used in the clinical practice and randomized studies [5, 19]. These indices were characterized by the presence of different items capturing the involvement of specific organs and systems including joints [5, 19]. In the SLEDAI-2K the item related to the joint involvement is then added to the others, returning a total score [5], thus avoiding the possibility of identifying the modification of this single manifestation. Indeed, the identification of joint involvement changes, in terms of improvement or worsening, is not possible by using the SLEDAI-2K [5]. Only the BILAG index permits reporting the modification of a specific manifestation, such as musculoskeletal involvement. However, this may not fully capture the changing overtime and has never been correlated with other disease parameters [19]. Moreover, in the clinical practice, the assessment of BILAG index needs extensive training [19].

A number of measures have been validated to assess disease activity in patients affected by RA, an inflammatory disease characterized primarily by joint involvement. Particularly, composite indices provide a comprehensive view of disease activity, by including the determination of the swollen/tender joints count, the physician's or patient's assessment, and the laboratory evaluation (ESR or C reactive protein) [20]. DAS28 is probably the most widely used since it is easy, quick, and standardized [6, 7].

This composite index has been applied in other rheumatic conditions, characterized by polyarticular involvement, such as PsA [8].

Thus, we used DAS28 to assess joint involvement in patients affected by SLE. Previous attempts to describe more accurately the SLE joint involvement have been described. In 2012, Islam et al. used the swollen/tender joint count, morning stiffness duration, visual analogue scale for articular pain, and physician's and patient's global assessment to evaluate the efficacy of the treatment with methotrexate and chloroquine on 41 SLE patients. A significant reduction of all considered parameters was observed after 24 weeks of follow-up [21]. More recently, a study conducted by Castrejõn et al. evaluated the application of Rheumatoid Arthritis Disease Activity Index (RADAI), a self-reported joint count, in patients affected by rheumatic diseases other than RA. Among these, 75 SLE patients were evaluated, showing the involvement of at least one joint in 59% of SLE patients [22].

In the present study, we found that the majority of SLE patients referred joint involvement showed DAS28 values between 3 and 5. Moreover, we could classify the activity status of the joint involvement according to DAS28 values, showing moderate/high disease activity in up to 60% of the cases and the presence of a remission only in 11.6%. The application of this categorization allows a better classification of the joint involvement in SLE patients in order to introduce the most appropriate treatment.

Nonetheless, most of the patients, classified without joint involvement when using the SLEDAI-2K (69.6%), had active disease defined by DAS28 and only 16.7% of them could be defined in DAS28 remission. This evidence suggests a low sensitivity of the SLEDAI-2K to capture joint involvement.

Moreover, to better ascertain the possible application of DAS28 in the assessment of SLE patients, a comparison with a group of RA patients was performed. Particularly, we aimed to discriminate the possible influence of ESR and GH in the determination of DAS28 values. No significant differences in terms of ESR and GH values were found between the SLE and RA patients. As known, ESR could be influenced by several SLE and non-SLE-related conditions. In our cohort, apart from three patients with kidney involvement, no other potentially ESR-influencing conditions, such as infections, were detected. This evidence suggests that the main manifestation potentially influencing the ESR values was musculoskeletal involvement itself. Similar considerations can be done about the GH, further confirmed by the agreement in terms of mean GH values between SLE and RA patients. Finally, significant differences were found when considering the mean values of swollen and tender joints count, which were significantly higher in RA patients, indicating, as expected, a more severe involvement in this group of patients.

In conclusion, the present study suggests the possibility of using the DAS28 index in the assessment of joint involvement in patients affected by SLE. This seems more sensitive compared with the global index SLEDAI-2K. Further longitudinal studies, with larger population and follow-up, enrolling patients at the beginning of the treatment, are needed to better clarify this issue.

## Figures and Tables

**Table 1:** Main demographic, clinical, laboratory, and therapeutic features of the SLE patients (*N* = 69) enrolled in the present study.

	Disease history	At the time of enrolment
Clinical manifestations (%)		
Skin involvement	59.4	20.3
Oral ulcers	26.1	2.8
Serositis	24.6	4.3
Hematologic disorder	40.6	27.5
Kidney involvement	26.1	4.3
Neurologic involvement	5.8	0
Laboratory evaluation (%)		
Anti-nuclear antibodies	100	94.2
Anti-double stranded DNA	73.5	34.8
Anti-Sm	35	24.6
Anti-SSA	49.2	49.3
Anti-SSB	20.2	7.2
Anticardiolipin IgG and/or IgM	46.4	17.4
Anti-*β*2-glycoprotein I IgG and/or IgM	27.5	7.2
Lupus anticoagulant	27.5	7.2
Low C3 and/or C4 serum levels	65.2	52.2
Drugs (%)		
Hydroxychloroquine	97.1	76.8
Methotrexate	34.8	8.7
Azathioprine	34.8	11.6
Mycophenolate mofetil	28.9	17.4
Cyclosporin A	33.3	10.1
Salazopyrine	1.4	0
Rituximab	5.9	0
Cyclophosphamide	10.1	0
Steroid dosage (mg/week, median, range)	—	35 (0–175)

**Table 2:** Comparison of disease activity parameters and status according to SLEDAI-2K definition of articular involvement (Group 1—patients with joint involvement defined by SLEDAI-2K, Group 2—patients without joint involvement defined by SLEDAI-2K).

	Group 1 (*N* = 21)	Group 2 (*N* = 48)	*P*
SLEDAI-2K (mean ± SD)	4.9 ± 1.3	1.5 ± 2.2	0.0001
Tender joint count (mean ± SD)	9.1 ± 5.0	3.6 ± 4.4	0.0001
Swollen joint count (mean ± SD)	4.6 ± 2.3	0.3 ± 0.6	0.0001
GH (mean ± SD)	58.0 ± 29.1	39.7 ± 22.8	0.01
ESR (mm/h, mean ± SD)	43.2 ± 32.8	23.2 ± 17.7	0.01
DAS28 (mean ± SD)	5.3 ± 1.2	3.5 ± 1.1	NS
DAS28 remission (*N*/%)	0/0	8/16.7	NS
DAS28 low disease activity (*N*/%)	1/4.7	21/43.7	0.001
DAS28 moderate disease activity (*N*/%)	6/28.6	23/47.9	NS
DAS28 high disease activity (*N*/%)	14/66.6	4/8.4	0.0001

**Table 3:** Mean ± SD of DAS28 and its component in the SLE (*N* = 69) and RA patients (*N* = 44).

	SLE (*N* = 69)	RA (*N* = 44)	*P*
ESR (mm/h, mean ± SD)	29.3 ± 24.9	32.7 ± 25.3	NS
Tender joint count (mean ± SD)	5.3 ± 5.2	15.7 ± 11.0	0.0002
Swollen joint count (mean ± SD)	1.6 ± 2.4	7.3 ± 2.4	0.0001
GH (mean ± SD)	45.3 ± 26.1	59.6 ± 23.8	NS
DAS28 (mean ± SD)	4.0 ± 1.4	5.5 ± 1.2	NS
